# News and Miscellany

**Published:** 1903-12

**Authors:** 


### News and Miscellany.

THE South Texas Medical Association meets in Galveston December 8th and 9th.

---

THE annual meeting of the Austin District Medical Society and election of officers will be held in Austin December 22nd. Full and interesting program, and a good supper.

---

The danger o'er, the patient is delighted;
God is forgotten and the doctor slighted.

"Why does the poor man gasp, papa,
Why does he gasp for breath?"
"He's heard of a man with a public job
Who worked himself to death."

---

According to an Austrian professor, water may be sterilized and made harmless and palatable all in five minutes by using the following solution, adding three drops to a gallon of water. Water 100 parts, potassium bromide 20 parts; then after five minutes add 3 drops of a nine per cent solution of ammonia.

---

Dr. Gartner, of Vienna, has just patented an instrument which records the pulse of a patient under the influence of anesthetic. The instrument is fastened to the forearm and a graduated disc records the increase or retardation of the pulse. The experiments in the hospital of Vienna succeed marvelously. It is hoped by means of it to prevent death during operations.

---

From the *Medical Age* we cull the following narrative, which is said to have occurred in Philadelphia: Miss Death, the daughter of an undertaker, suffered from appendicitis and was operated upon by Dr. Dye who placed her in charge of two nurses, namely, Miss Payne by day and Miss Grone by night. In spite of the gruesome combination the patient made a rapid recovery."

---

The Perils of Microbe-Dodging.—"Microbes snatch at us from around every corner," according to Eugene Wood in the November "Everybody's." "We can get on the good side of a dog by patting his head, and we can please the cat by scratching her under the chin (if she doesn't scratch first). We can tame other animals by giving them food or by putting the weight of our hand on them. If they won't be petted or tamed we can pick up a rock and let them have it between the eyes. But when a creature has no tail to wag and nothing to purr with, how can we pet it? How can we, without getting a crick in the neck, stoop down far enough to say "Pretty Microbe!" to something that is to us as a grain of sand is to Mount Blanc. If it comes to exterminating them, what chance have we with a creature that every two hours breaks into two pieces, each of which is a perfect organism, ready in another two hours to break into two again, and each of these halves to break in two in another two hours, and so on and so on until in three days the progeny of one single bacterium numbers 4,772,000,000,000? Nobody can keep up with that rate of increase. Of all the discoveries made by science it seems to me that the most disheartening is the discovery of germs."—*Medical Herald.*

---

DEATH OF DR. WARREN.—Dr. William Matthew Warren, general manager of the great business of Parke, Davis & Co., died at his home in Detroit, November 11, ult., age 39 years. The directors, executives and employes of the business met and paid a beautiful and merited tribute to his memory, and have issued it in connection with an obituary. Dr. Warren entered the service of Parke, Davis & Co. when only 17 years of age. He rose by real worth to be general manager, the highest position in the gift of the house, which position he had filled seven years to the date of his death. The JOURNAL is in receipt of the sad news just as we go to press.

---

DR. J. W. TALBOT, Texarkana, Texas, will receive for treatment and give personal attention to cases of morphine, cocaine and chloral addiction.

---

WILL REPAIR YOUR ELECTRICAL MACHINES.—Static and all electrical medical apparatus put in running order. I am also agent for electrical and X-ray apparatus. Oliver Brush, 710 Colorado Street, Austin, Texas.

---

NEW ORLEANS POLYCLINIC.—Seventeenth annual session opens November 2, 1903, and closes May 28, 1904. Physicians will find the Polyclinic an excellent means for posting themselves upon modern progress in all branches of medicine and surgery. The specialties are fully taught, including laboratory work. For further informaton, address New Orleans Polyclinic, postoffice box 797, New Orleans, La.

---

THE DAILY MEDICAL JOURNAL will be published January 1, 1904. We need a physician as staff correspondent in every town in this State, to supply us with scientific, social, institutional and personal news, and will pay regular newspaper rates for this service. Instructions, stationery, and badge free. Address Mr. J. Antonowvitsch, 154 East Seventy-second street, New York City.

---

The Patriarchs.—Our young friends, Drs. Bowers and Harrison, celebrated their anniversaries this week—the one 86 and the other 77 years of age—and both still in harness. May each reach a hundred.—*Columbus* (Tex.), *Herald.* Dr. J. M. Reuss of Cuero, Dr. Jo H. Reuss' father, and an old friend of the "Red-Back," celebrated his eightieth birthday December 12th, inst. The *Cuero Star* produced a picture of the venerable doctor, who is yet hale and hearty, and gave him a pretty editorial writeup.

---

Special Offer.—I would call attention to the Upjohn Co.'s special offer (see advertisement). Drop them a postal card mentioning this, and you will hear from them. Their goods are recognized everywhere as standard. They give $5 for $1.

---

In one of the most embarrassing positions possible, one which required infinite tact and self-control, Dr. Tabor has proven himself a man among men. His honesty and impartial consideration of every question concerning the quarantine at San Antonio and Laredo have won the respect of those who have differed with him most widely in regard to his opinions, and all concede his desire to do what is for the best interests of the State.—*State Topics.*

Yes, Dr. Tabor has shown himself to be the right man in the right place. Governor Lanham is to be congratulated upon the judiciousness of his seclection of Dr. Tabor for the high, responsible and difficult position of State Health Officer, and the whole State of Texas is likewise to be congratulated that they have, as head of the Public Health Department, a man of courage, intelligence and zeal. To his sleepless vigilance and knowledge of sanitary science, and the courage to buck against commercial interests for the protection of the public health, and face unjust criticism, is due our escape from a devastating epidemic.

---

Memorial Hospital.—At Richmond, Va., there has been built a magnificent hospital called the "Charlotte Williams Hospital,"

and also "Memorial Hospital." It is equipped in the most thorough manner. John L. Williams contributes $50,000 to the endowment fund. Dr. J. S. Horsley, recently of El Paso, Texas, and now of the faculty of the Virginia Medical College, and on the surgical staff of the hospital, contributes $5,000. There are a number of "funds" beside the endowment. The "Memorial Ward Building Fund," "Sustenance Fund" (to which the Medical College of Virginia gives yearly $6000) and the "Operating Room Fund." No profit is to be derived from the hospital.

---

Dr. J. W. Hudson of Milano, in "being good" and "doing it now," writes that the tear as big as a biscuit that I dropped when Dr. Bigike beat me out of five years subscription wasn't a circumstance to the one he dropped when he read "Doctors and Doctors," in the November number.

---

Quarantine against Laredo was raised November 30th, ult., after freezing weather. Total cases reported to that date, 1028; total deaths, 97.

---

The East Texas Medico-Chirurgical Society met in Palestine November 19th and 20th, with a full program, which was carried out, after which the society reorganized and began work under the new constitution and by-laws, which provides that the word association be used instead of society, and that the officers be elected in November instead of May, and the following officers were elected: E. W. Link, M. D., President, Palestine; John H. Joyce, M. D., Vice-President, Buffalo; F. B. Moore, M. D., Treasurer, Palestine; E. E. Guinn, M. D., Secretary, Jacksonville.

---

Now the hurly-burly's done, now the battle's lost and won, comes the aftermath. Our San Antonio letters, to which so large a part of this issue is devoted, furnish interesting reading and constitute a permanent record.

---

Death of Dr. Murray.—It will be remembered that Dr. R. D. Murray, the venerable Marine Hospital Service surgeon and yellow fever expert, was thrown from a carriage in Laredo some weeks ago and badly hurt. He died of pneumonia resulting from his injuries November 22d, ult. Dr. Murray entered the Marine Hospital Service in 1872. He was a native of Ohio, and in his sixty-fifth year. The remains were interred at Bluffton, Ohio, the home of his son.

---

Dr. Tabor will go to Washington on the 6th inst. on official business, and return on the 20th. The Austin District Medical Society will meet on the 22nd and "yellow fever" will be served, red hot, I presume. Dr. Tabor and some of the San Antonio and Galveston doctors are expected to be present. Annual election of officers.

---

The Late Medical Director of Confederate States Hospitals—Some Remarkable Figures.—In the *Picayune* of November 3rd (ult.) is published a letter from Dr. C. H. Tebault, Surgeon-General United Confederate Veterans, to Mrs. S. H. Stout, Clarendon, Texas, in which Dr. Tebault, after expressing sentiments of the tenderest sympathy with the family in the loss of their distinguished husband and father and paying a beautiful and merited tribute to Dr. Stout's worth and work, says:

"It was to be expected that such people as constituted the South should have cared for with tender hearts and with their best efforts all prisoners of war in their midst, and such was the case. From first to last the Confederates held of Federal prisoners 270,000, and the Federals held of Confederate prisoners 220,000. Thus, with an excess of 50,000 more Federal prisoners, there died in Confederate hospital service 4000 less Federal prisoners than the Federals lost of Confederates in their hospital service.

"This historic fact in relation to the prisoners of war held by the Confederates must have proved a source of great consolation to your good husband and our loved comrade at the last supreme moment to feel and know how largely his own humanitarian administration had contributed to this superior result in behalf of the helpless Federal prisoners in our keeping, whom their own government persistently refused to exchange for our Confederates, while every possible effort on our part was made to obtain such exchange.

"Much more could be written of the splendid humanitarian work done and accomplished under the brilliant administration of Confederate hospitals under your husband and our comrade, but enough has been presented to challenge the attention of the historian, and for preservation by his children and grandchildren. With failing words to convey my own personal sorrow and abiding sympathy for you and for yours, I am, sincerely your friend."

---

Dr. Geo. J. Engelmann, the distinguished gynecologist, died November 16th, ult., aged 55.

